# Association between Apnea of Prematurity and Respiratory Distress Syndrome in Late Preterm Infants: An Observational Study

**DOI:** 10.3389/fped.2016.00105

**Published:** 2016-09-26

**Authors:** François Olivier, Sophie Nadeau, Georges Caouette, Bruno Piedboeuf

**Affiliations:** ^1^Service of Neonatology, Department of Pediatrics, CHU de Québec-Université Laval, Quebec, QC, Canada

**Keywords:** apnea, respiratory distress syndrome

## Abstract

**Background:**

Late preterm infants (34–36 weeks’ gestation) remain a population at risk for apnea of prematurity (AOP). As infants affected by respiratory distress syndrome (RDS) have immature lungs, they might also have immature control of breathing. Our hypothesis is that an association exists between RDS and AOP in late preterm infants.

**Objective:**

The primary objective of this study was to assess the association between RDS and AOP in late preterm infants. The secondary objective was to evaluate if an association exists between apparent RDS severity and AOP.

**Methods:**

This retrospective observational study was realized in a tertiary care center between January 2009 and December 2011. Data from late preterm infants who presented an uncomplicated perinatal evolution, excepted for RDS, were reviewed. Information related to AOP and RDS was collected using the medical record. Odds ratios were calculated using a binary logistic regression adjusted for gestational age and sex.

**Results:**

Among the 982 included infants, 85 (8.7%) had an RDS diagnosis, 281 (28.6%) had AOP diagnosis, and 107 (10.9%) were treated with caffeine for AOP. There was a significant association between AOP treated with caffeine and RDS for all infants (OR = 3.3, 95% CI: 2.0–5.7). There was no association between AOP and RDS in 34 weeks infants [AOR: 1.6 (95% CI: 0.7–3.8)], but an association remains for 35 [AOR: 5.7 (95% CI: 2.5–13.4)] and 36 [OR = 7.8 (95% CI: 3.2–19.4)] weeks infants. No association was found between apparent RDS severity and AOP, regarding mean oxygen administration duration or complications associated with RDS.

**Conclusion:**

The association between RDS and AOP in late preterm infants reflects that patients affected by RDS are not only presenting lung immaturity but also respiratory control immaturity. Special consideration should be given before discontinuing monitoring after RDS resolution in those patients.

## Introduction

Late preterm infants, born between 34^0/7^ and 36^6/7^ weeks gestational age (GA), account for an important proportion of newborns requiring neonatal intensive care, mainly because of respiratory morbidity (1–5). As an example, they have a significantly higher risk of respiratory distress syndrome (RDS) than term infants (RR: 17.3; 95% CI, 9.8–30.6) (4). The incidence of RDS in late preterm infants was 15.4% in a study realized by the Canadian Neonatal Network (CNN) (6). Late preterm infants affected by RDS typically exhibit respiratory distress progressing over 48–72 h, associated with symptoms, such as tachypnea, nasal flaring, retraction, grunting, and hypoxemia. Apnea of prematurity (AOP) is also a more frequent diagnosis in late preterm than in term infants, affecting up to 10% of those born at 34 and 35 weeks (4, 7, 8). Since AOP is rarely objectified in the first few days of life, the diagnosis could easily be omitted with only a short period of monitoring (9, 10). Late preterm infants are also recognized to be at higher risk of unfavorable neurodevelopmental outcomes when compared with term infants (11–15). Developmental impacts of AOP are not well-known, and a causal relationship is not clearly established. However, some data raise great concerns about untreated AOP and neurodevelopmental impairments (16–19). As a consequence, pediatricians are daily confronted with monitoring issues and safe discharge concerning those infants. As AOP is a manifestation of immaturity, it might be worthwhile to pay attention to other immaturity manifestations, as they may be associated with AOP. This would help target more precisely which patient needs monitoring. We postulate that as late preterm infants affected by RDS have immature lungs, they may also have immature control of breathing and are at higher risk of AOP. Therefore, we hypothesized that RDS will be associated with AOP in late preterm infants. The primary objective of this study was to assess the association between RDS and AOP in a late preterm population. The secondary objective was to evaluate the association between apparent RDS severity and AOP in the population of late preterms affected by RDS.

## Materials and Methods

### Study Design and Population

**Figure 1:**
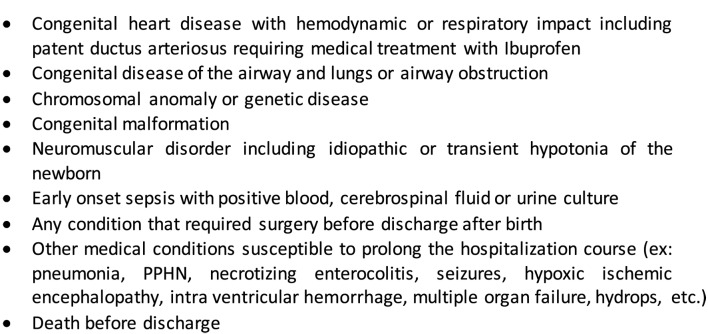
**Exclusion criteria**.

This is a tertiary care center, retrospective cohort study of the population of late preterm infants admitted between January 2009 and December 2011. Participants were identified through medical records review. The studied population included late preterm infants born or admitted to the Mother and Child Center of the CHU de Québec and who presented an uncomplicated perinatal evolution, except for RDS. We included only patients susceptible to be discharged soon after birth or after RDS resolution. The list of every diagnosis from the medical records was reviewed for each patient by the main author. Those who presented any medical or surgical condition that could significantly prolong the need for cardiorespiratory monitoring were excluded according to pre-specified criteria (Figure 1). The included patients were hospitalized either in the neonatal intensive care unit (NICU), in the newborn nursery, or in the moms’ room. Frequent reasons for NICU admission at birth included respiratory distress and birth weight under 2.3 kg as per our protocol. All the patients admitted to the NICU received systematic cardiorespiratory monitoring. Those requiring intravenous infusion were hospitalized in the newborn nursery. This study was approved by the hospital ethics committee. Since this is a retrospective study and that all information collected were anonymous, no consent was required.

### Variables and Data Sources

The primary outcomes of this study were AOP, and AOP treated with caffeine (caffeine citrate). Patients were reported having AOP and/or RDS diagnosis if these diagnoses were reported on the medical discharge summary. The diagnosis of AOP and RDS was confirmed on discharge summary by the attending physician who sent the patient home. To be classified as having a RDS, the patient must have required respiratory support and being hospitalized in the NICU. In the institution, AOP is defined by one of these three situations: the absence of breathing movements for 15 s or more, an abnormal respiratory pattern (bradypnea or superficial respiration) associated with oxygen saturation below 85%, or bradycardia below 100 beats per minute (20). Distinction between central, obstructive, or mixed apnea was not possible. Caffeine treatment was documented according to the hospital’s pharmacy database. Caffeine administration was based on clinical decision of the attending physician, according to frequency or severity of AOP, without pre-established criteria. GA and sex were considered as potential confounding factors for the main outcomes and were obtained from the medical records. Information from medical records and from pharmacy databases was merged according to chart numbers.

Secondary outcomes were only assessed in patients affected by RDS whether they presented AOP and/or received caffeine treatment or not. They included the duration of oxygen administration, ventilation support, and NICU hospitalization, as well as the combined outcome of mechanical ventilation exposure and pneumothorax. Ventilatory support included mechanical ventilation, non-invasive respiratory support/ventilation (nasal continuous positive pressure ventilation, nasal intermittent positive pressure ventilation, and sight positive airway pressure), and high flow nasal cannula. The charts of patients with RDS diagnosis have been reviewed to collect the data related to the secondary outcomes.

### Bias

As AOP diagnosis were made clinically, without diagnostic evaluation for most of the patients, the outcome of AOP treated with caffeine was used as a proxy for infants with more significant AOP. This strategy was used in order not to overestimate the association between RDS and AOP.

### Statistical Methods

Descriptive statistics are presented using percentages and means with SD. Adjusted analysis for sex and GA is presented using mean and SE. The primary outcomes were tested using a binary logistic regression model to obtain odds ratio. The simple effects between sex, GA, and RDS on AOP were assessed, and interaction was tested when necessary. Secondary outcomes were analyzed using an ANOVA, estimated with a linear mixed model for heterogeneous variances, to see the effects of caffeine or apnea, and adjusted for GA and sex. Bonferroni multiplicity correction was used when needed. All statistical analyses were performed with SAS, version 9.3 (SAS Institute Inc., Cary, NC, USA).

## Results

During the study period, 1101 late preterm infants were admitted to the Mother and Child Center of the CHU de Québec and 119 (10.8%) were excluded according to the exclusion criteria (Appendix A). Among all excluded patients, there were 35, 40, and 44 patients, respectively, aged from 34 to 36 weeks GA. Twenty (16.8%) of them had a RDS diagnosis, and 37 (31.1%) had an AOP diagnosis. Fifteen (12.6%) had both AOP and RDS diagnosis.

Among the 982 patients forming the studied group, 85 (8.7%) had a RDS diagnosis, 281 (28.6%) had an AOP diagnosis, and 107 (10.9%) were treated with caffeine for AOP. Patients with RDS diagnosis were all admitted to the NICU and required respiratory support. Among the 897 patients not affected by RDS, 65% were admitted in the NICU.

Distribution according to GA and sex and descriptive data for the primary outcomes are presented in Table 1. The proportion of males increased from 34 to 36 weeks in the RDS group, but it remained stable in patients without RDS (Table 1). In the group of patients with RDS, 21 were exposed to mechanical ventilation and also received surfactant and 11 presented a pneumothorax.

**Table 1:** **Study population**.

Patients included in the study (*N* = 982)	Patients with RDS (*N* = 85)	Patients with no RDS (*N* = 897)
**Mean birth weight, grams (SD)**	2523 (386)	2504 (435)
**GA, *N* (%)**		
**34^0/7^ to 36^6/7^**	85 (100%)	897 (100%)
34^0/7^ to 34^6/7^	35 (41.1%)	191 (21.3%)
35^0/7^ to 35^6/7^	27 (31.8%)	244 (27.2%)
36^0/7^ to 36^6/7^	23 (27.1%)	462 (51.5%)
**Males, *N* (%)**		
**34^0/7^ to 36^6/7^**	52/85 (61.2%)	465/897 (51.9%)
34^0/7^ to 34^6/7^	16/35 (45.7%)	106/191 (55.2%)
35^0/7^ to 35^6/7^	18/27 (66.7%)	123/244 (50.6%)
36^0/7^ to 36^6/7^	18/23 (78.3%)	236/462 (51.2%)
**AOP, *N* (%)**		
**34^0/7^ to 36^6/7^**	54/85 (63.5%)	227/897 (25.3%)
34^0/7^ to 34^6/7^	27/35 (77.1%)	129/191 (67.5%)
35^0/7^ to 35^6/7^	18/27 (66.7%)	63/244 (25.8%)
36^0/7^ to 36^6/7^	9/23 (39.1%)	35/462 (7.6%)
**Caffeine, *N* (%)**		
**34^0/7^ to 36^6/7^**	22/85 (25.9%)	85/897 (9.5%)
34^0/7^ to 34^6/7^	15/34 (42.9%)	50/191 (26.2%)
35^0/7^ to 35^6/7^	4/27 (14.8%)	26/244 (10.7%)
36^0/7^ to 36^6/7^	3/23 (13.0%)	9/462 (1.9%)

### Primary Outcomes

#### Apnea of Prematurity

There was a significant interaction between RDS and GA for the AOP outcome (*p* = 0.015). Therefore, AOP odds ratio were stratified by GA, and there was no more association between RDS and AOP in 34 weeks infants (OR = 1.6, 95% CI: 0.7–3.7). Nonetheless, a significant association remains for 35 (OR = 6.2, 95% CI: 2.6–14.6) and 36 (OR = 8.8, 95% CI: 3.5–22.0) weeks infants. The overall AOP rate was 30.0% in females and 27.1% in males. Therefore, being a male was slightly associated with a decreased risk of AOP (OR = 0.69, 95% CI: 0.49–0.97) according to the logistic regression model. There was no interaction between GA and sex (*p* = 0.33) neither between RDS and sex (*p* = 0.27) for the AOP outcome.

#### AOP Treated with Caffeine

Both GA (OR = 3.13, 95% CI 1.94–5.06, *p* < 0.0001) and RDS (OR = 2.24, 95% CI 1.26–3.97, *p* = 0.0059) were individually associated with caffeine exposure according to the logistic regression model. There was no interaction between GA and RDS for that issue (*p* = 0.13). RDS was associated with AOP treated with caffeine (OR = 3.3, 95% CI: 2.0–5.7). There was no interaction between GA and sex (*p* = 0.97) neither between RDS and sex (*p* = 0.66) for the outcome of being treated with caffeine for AOP.

### Secondary Outcomes

Results of the secondary outcomes for the 85 patients affected by RDS are summarized in Table 2 and Table 3.

**Table 2:** **Secondary outcomes summary for patients with RDS regarding AOP**.

AOP, Yes (54)/No (31)	Unadjusted mean^a^ (SD)	Adjusted mean^a^ (SE)	ANOVA *p*-value
Oxygen duration (days)	2.3 (1.7)/2.1 (2.0)	2.5 (0.2)/2.1 (0.3)	0.3
Ventilation (days)	5.4 (3.0)/3.7 (1.9)	5.2 (0.3)/4.0 (0.4)	0.02
Hospitalization (days)	12.5 (8.2)/8.4 (3.3)	12.3 (1.1)/8.9 (0.7)	0.01

*^a^No interaction where significant for GA and sex and therefore the model used does not consider it*.

**Table 3:** **Secondary outcomes summary for patients with RDS regarding AOP treated with caffeine**.

Caffeine for AOP, Yes (22)/No (63)	Adjusted mean^a^ (SE)	ANOVA *p*-value	ANOVA *p*-value
Oxygen duration (days)	1.5 (1.4)/2.5 (1.9)	1.8 (0.4)/2.6 (0.2)	0.08
Ventilation (days)	5.4 (3.6)/4.6 (2.4)	5.4 (0.5)/4.6 (0.3)	0.2
Hospitalization (days)	14.9 (10.6)/9.7 (4.7)	14.6 (2.4)/9.8 (0.6)	0.05

*^a^No interaction where significant for GA and sex and therefore the model used does not consider it*.

#### Oxygen

Mean oxygen duration for RDS was 2.3 (SD: 1.8) days for all patients. No significant relation was found between oxygen duration for RDS and AOP or caffeine administration for AOP.

#### Ventilatory Support

Mean ventilation support duration was 4.8 (SD: 2.8) days for all patients. Apnea diagnosis was associated with prolonged ventilation support, but this difference was not significant when addressing patients treated with caffeine.

#### NICU Hospitalization

Mean hospitalization duration in the NICU was 11.4 (SD: 7.1) days for all patients, and it was significantly longer in patients who presented AOP. The same association was observed for patients treated with caffeine, but the difference was borderline significant.

#### RDS Complications

No association was found in the regression model between AOP and the composite outcome of pneumothorax and/or invasive ventilation exposure (OR = 0.35, 95% CI: 0.12–1.03, *p* = 0.06). This composite outcome was nonetheless associated with prolonged oxygen duration (3.1 vs. 2.1 days, *p* = 0.02), but no association was found with ventilation support duration (4.6 vs. 5.1 days, *p* = 0.34) neither hospitalization duration (10.8 vs. 11.6 days, *p* = 0.61).

## Discussion

This study supports that late preterm infants suffering of RDS are at higher risk of AOP. Therefore, it confirms our research hypothesis regarding the association between respiratory manifestations of immaturity and AOP in late preterm infants. RDS was associated with AOP in 35 and 36 weeks infants, which are normally expected to show a higher level of respiratory control maturity, but not in 34 weeks infants. It is possible that patients of 34 weeks had a similar AOP rate in both groups because they have been more systematically monitored, even those without RDS. However, the association between AOP treated with caffeine and RDS was significant for all infants without interaction with GA. In fact, this association reflects the link between RDS and more significant AOP.

In 2011, Eichenwald et al. published a study addressing AOP very similar to our regarding inclusion criteria and data sources, but targeting 33 and 34 weeks’ infants instead of late preterm (21). Therefore, the AOP incidence reported in their study (49%) was superior to the one found in our study (29%). Interestingly, they found a similar relation between AOP and length of hospital stay (21). In our study, there was no association either between duration of oxygen supplementation or RDS complications and the occurrence of AOP. This suggests that there is no relation between the severity of RDS and AOP. Nonetheless, we observed a significant interaction between duration of ventilation support and AOP in the RDS group. As there is no relation between oxygen supplementation and AOP, we hypothesized that the longer maintenance of ventilation support in that group was related to AOP rather than RDS.

Different mechanisms may contribute to the association between RDS and AOP in the population of late preterm infants. First, important changes in the dynamic of lung compliance occur until 38 weeks, contributing to the stabilization of the residual functional capacity (1, 22). The decreased in the residual functional capacity observed in RDS increase the susceptibility to more frequent and prolonged desaturations (1). Furthermore, RDS is thought to affect autonomic control in late preterm infants since it is associated with long-term decrease in heart rate variability, a pattern usually observed in more preterm infants. The mechanism explaining this autonomic variation needs to be better understand, but it may be related to RDS itself and could account for persistent AOP (8, 23). Otherwise, it is known that late preterm infants have an immature respiratory response to hypoxia and to hyperoxia (22, 24). Dysregulation of peripheral chemoreceptors sensitivity, due to early postnatal hypoxemia, may be involved in the increase susceptibility to AOP in infants affected by RDS (24). Furthermore, chemoreceptors effectiveness remains dependent to the response of central mechanisms varying with the degree of brain maturation, which is not completed in late preterm infants (24). Additionally, the decline in ventilatory response associated with hyperoxia is known to increase the frequency of AOP, especially in those that exhibit periodic breathing, a frequent respiratory pattern present in late preterm infants (22, 25). This could have been an issue in patients exposed to prolonged oxygen administration for RDS, beyond the first days of life.

Clinicians’ concerns about AOP are mainly related to hypoxic episodes and their impact on long-term neurological development. Although they are not clearly supported by the literature, potential life threatening events are also worrisome. It is known that late preterm infants are at higher risk of unfavorable neurodevelopmental outcomes when compared with term infants (11–15). Even though the causal relation between AOP and poor neurodevelopmental outcome is not clearly established, some data raise great concerns about this issue, mainly in extremely preterm infants (16, 17, 19, 26, 27). Therefore, the optimal duration of cardiorespiratory monitoring in accordance with the natural evolution of AOP is unknown. Nonetheless, it is suggested that the first days of life are not the best window to proceed to a respiratory control diagnostic study (respirogram or pulse oximeter recording) (9). In our center, no infant is submitted to such an evaluation before 7 days of life and usually not before 39 weeks corrected age. Once an infant present AOP, he will remain on monitoring for up to 7 days after its last spell. Because cardiorespiratory monitoring is not mandatory, it may be discontinued shortly after RDS resolution if no AOP is documented during the initial monitoring. Evaluation is only requested for infants who present clinical manifestations of apnea, and no monitoring of the saturation in the car seat is performed in our institution before discharge as per new recommendations in Canada (28, 29).

Although our study included a large number of late preterm infants, we must acknowledge some of its limitations. On the one hand, the diagnosis of AOP was made clinically, and this may have underestimated the real incidence of AOP, mainly in 35 and 36 weeks infants without RDS. These patients were probably less likely to be monitored, while patients affected by RDS were systematically monitored. If this hypothesis is exact, it may have resulted in an increased likelihood of the association between AOP and RDS. On the other hand, we may have overestimated the global rate of AOP because of the diagnosis criteria used in our center. Indeed, AOP diagnostic criteria remained variable between institutions as the cut off for bradycardia may vary from 80 to 100. Eichenwald et al. (21) reported this issue in their study pointing out the subjectivity of the diagnosis and the accuracy of data about AOP. They reported a rate of AOP diagnosis ranging from 24 to 76% among 10 sites. In our study, the 100 cut off point for bradycardia made the diagnosis more likely and therefore we may have overestimated the rates of AOP. Nonetheless, the same diagnosis criteria were used for all infants, and it should not have impacted the association between RDS and AOP. Furthermore, using the odds ratio for caffeine treatment allows to get a more significant picture from a clinical standpoint as patients treated with caffeine certainly presented more important manifestations of AOP either in severity or frequency. In rare cases, patients may have received caffeine before being discharged with home monitoring for periodic breathing, and this study did not allow us to identify those patients. However, periodic breathing is a pattern of alternating breaths and brief respiratory pauses and is considered as a marker of AOP (30).

## Conclusion

Our study supports that RDS, a manifestation of pulmonary immaturity, is a marker for higher susceptibility of respiratory control immaturity in late preterm infants since it is associated with AOP. Therefore, special consideration should be given before discontinuing monitoring after RDS resolution. A prospective study addressing respiratory control would be an important step toward achieving clarity on the issue of AOP in late preterm infants. It could also allow to determine the best window and the appropriate duration for cardiorespiratory monitoring.

## Author Contributions

FO: wrote the manuscript, revised, and approved the final manuscript as submitted. SN: reviewed and revised the manuscript and approved the final manuscript as submitted. GC: reviewed and revised the manuscript and approved the final manuscript as submitted. BP: reviewed and revised the manuscript and approved the final manuscript as submitted.

## Conflict of Interest Statement

The responsible author, FO, has no disclosure. The other authors have no conflicts of interest to disclose. The authors declare that the research was conducted in the absence of any commercial or financial relationships that could be construed as a potential conflict of interest.

## Abbreviations

AOP, apnea of prematurity; GA, gestational age; NICU, neonatal intensive care unit; RDS, respiratory distress syndrome.

## Appendix

### A. 119 Excluded patients per categories

1. Congenital heart disease with hemodynamic or respiratory impact including patent ductus arteriosus requiring medical treatment with Ibuprofen
  ◦ 7 patients
2. Congenital disease of the airway and lungs and airway obstruction
  ◦ 6 patients
3. Chromosomal anomaly and genetic disease
  ◦ 11 patients
4. Congenital malformation
  ◦ 26 patients
5. Neuromuscular disorder including idiopathic or transient hypotonia of the newborn
  ◦ 6 patients
6. Early onset sepsis with positive blood, cerebrospinal fluid, or urine culture
  ◦ 7 patients
7. Any condition that required surgery before discharge after birth
  ◦ 16 patients
8. Other medical conditions susceptible to prolong the hospitalization course (e.g., pneumonia, PPHN, necrotizing enterocolitis, seizures, hypoxic ischemic encephalopathy, IVH, multiple organ failure, and hydrops)
  ◦ 39 patients
9. Death before discharge
  ◦ 1 patient
